# Electrical stimulation via repeated biphasic conducting materials for peripheral nerve regeneration

**DOI:** 10.1007/s10856-023-06763-x

**Published:** 2023-11-15

**Authors:** Tabitha N. Rosenbalm, Nicole H. Levi, Michael J. Morykwas, William D. Wagner

**Affiliations:** 1 School of Biomedical Engineering and Sciences, Wake Forest University-Virginia Polytechnic Institute and State University, Winston-Salem, NC 27106 USA; 2 Department of Plastic and Reconstructive Surgery, Wake Forest Baptist Health, Winston-Salem, NC 27157 USA

## Abstract

Improved materials for peripheral nerve repair are needed for the advancement of new surgical techniques in fields spanning from oncology to trauma. In this study, we developed bioresorbable materials capable of producing repeated electric field gradients spaced 600 μm apart to assess the impact on neuronal cell growth, and migration. Electrically conductive, biphasic composites comprised of poly (glycerol) sebacate acrylate (PGSA) alone, and doped with poly (pyrrole) (PPy), were prepared to create alternating segments with high and low electrically conductivity. Conductivity measurements demonstrated that 0.05% PPy added to PSA achieved an optimal value of 1.25 × 10^−4^ S/cm, for subsequent electrical stimulation. Tensile testing and degradation of PPy doped and undoped PGSA determined that 35–40% acrylation of PGSA matched nerve mechanical properties. Both fibroblast and neuronal cells thrived when cultured upon the composite. Biphasic PGSA/PPy sheets seeded with neuronal cells stimulated for with 3 V, 20 Hz demonstrated a 5x cell increase with 1 day of stimulation and up to a 10x cell increase with 3 days stimulation compared to non-stimulated composites. Tubular conduits composed of repeated high and low conductivity materials suitable for implantation in the rat sciatic nerve model for nerve repair were evaluated in vivo and were superior to silicone conduits. These results suggest that biphasic conducting conduits capable of maintaining mechanical properties without inducing compression injuries while generating repeated electric fields are a promising tool for acceleration of peripheral nerve repair to previously untreatable patients.

**Graphical Abstract:**

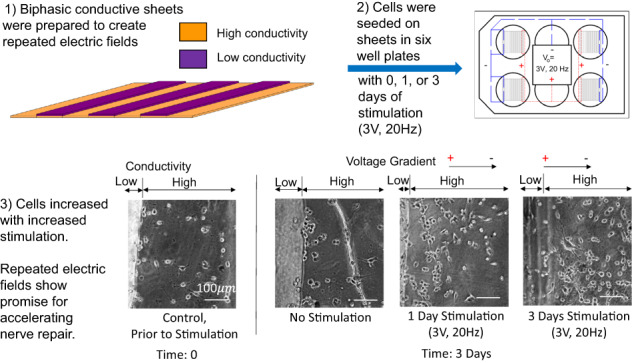

## Introduction

Each minute, the lives of ten people in the United States have been altered by a peripheral nerve transection [1]. While autografts have remained a common treatment, the drawbacks of donor site morbidity and limited availability have highlighted the need for off-the-shelf conduits [2, 3]. Research has transformed the early non-degradable silicone conduits to biodegradable materials tailored to match nerve’s mechanical properties. Unfortunately, about 87% of nerve injury patients have no treatment options due to age (>40 years) or nerve gap length (>3 cm). For these patients, slow nerve outgrowth coupled with rapid scar formation has been shown to prevent axonal extension to the distal target [2, 4]. Overwhelming patient need presents a ready market for next-generation conduits which mimic the structural, chemical, and/or electrical native nerve environment to promote and maintain axonal outgrowth for faster nerve recovery over longer distances.

Nerve repair research has focused on materials [3, 5–18], topography [18–31], molecular factors [8, 9, 11–14, 30–48], and electrical stimulation [5, 42, 46, 48–69] as potential triggers to promote and maintain axonal outgrowth. Among these stimuli, continuous 100μs pulse trains of electrical stimulation (3 V, 20 Hz for durations from 1 h and up to 2 weeks) across a transected nerve induced a three-fold increase in axonal outgrowth for up to 48 h [51]. To extend the time of accelerated axonal outgrowth, axonal responses to endogenous electrical stimulation were examined. In separate studies of bovine and rat corneas, epithelial cells produced electric fields across gaps between intact cells (with normal surface potential, 40 mV) and collapsed, damaged cells (effectively an electrical ground). These electric fields provided stimulation, which directed axonal growth across transected gap lengths less than 600 μm [56, 70]. Together, these data suggest that an electric field can promote and maintain axonal outgrowth across a limited distance (600 μm) [51, 56, 70].

**Fig. 1:**
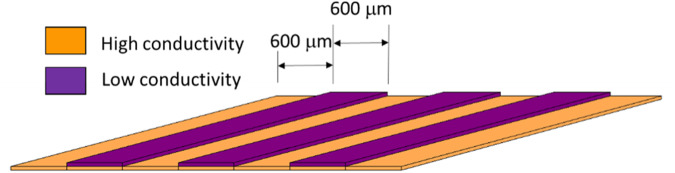
Schematic of Repeated Biphasic Conducting Sheets. High and low conductivity polymers alternated along the length of the sheets in 600 μm segment widths to enable production of repeated electric fields via ionic solution flow or implanted electrical stimulator

In this study, we have developed bioresorbable materials capable of producing repeated electric field gradients at the observed 600 μm distances to assess impact on nerve growth across a gap. To achieve this, high and low conductivity polymers (i.e., biphasic) were alternated (in 600 μm segments), such that, repeated electric fields could be generated by native ionic solution flow or a controlled implantable stimulator (Fig. 1). Ranges for elastic modulus and ultimate strain were determined based on mechanical properties of excised rat sciatic nerve and human ulnar nerve [71–73], human nerve healing time frame [74], and Ohm’s law applied to known electric field properties surrounding nerve tissue (Table 1). Poly (glycerol sebacate) acrylate (PGSA) was selected as the base material due to its crosslinking time, strength, flexibility, and degradation time [75–79]. PGSA was synthesized with varied acrylation (25, 30, 35, and 40%) to optimize the elastic modulus and ultimate strain. Soluble PPy was selected as a dopant for PGSA to achieve target high electrical conductivity properties to maximize composite conductivity while maintaining the same base material throughout the construct. Fibroblastic (HEPM) and neuronal (B35) cell lines were cultured on biphasic sheets to evaluate biocompatibility. B35 cells were applied further to estimate axonal response to the biphasic conducting sheets, without or with electrical stimulation (3 V, 20 Hz for 1 or 3 days). To our knowledge, this study provides the first report on the effects of repeated electrical field stimulation to accelerate healing of peripheral nerves.

**Table 1:** Mechanical and electrical target parameters for biphasic conducting tube [71–74]

Property	N/mmElastic Modulus [N/mm^2^]	Ultimate Strain [%]	Degradation Rate [months]	High Conductivity [S/cm]	Low Conductivity [S/cm]
Material Target Value	0.4–19.3	4–30	3–12	1.25 × 10^−4^	1.25 × 10^−5^

## Materials and methods

### Materials

Poly (glycerol sebacate) (PGS), poly (glycerol sebacate) acrylate (PGSA), and poly (pyrrole) (PPy) were synthesized in our laboratory. Pyrrole, acryloyl chloride, triethylamine, sebacic acid, dioctyl sulfosuccinate sodium salt, ammonium persulfate, chloroform, 2, 2 dimethyl-2-phenyl acetophenone, and chloroform-d were purchased from Sigma Aldrich (St. Louis, MO). Sylgard 184 silicone elastomer kit (Dow Corning, Greensboro, NC) was purchased from Ellsworth Adhesives (Germanton, WI). Glycerol, methylene chloride, and ethyl acetate were purchased from Fisher Scientific (Pittsburgh, PA). Fibroblastic (human embryonic palatal mesenchyme cells, HEPM, CRL-1486) and neuronal (rat neuronal neuroblast, B35, CRL-2754) cell lines were purchased from ATCC (Manassas, VA). Dulbecco’s Modified Eagle Medium (DMEM), sodium pyruvate, L-glutamate, D-glucose, penicillin streptomycin, and trypsin were purchased from Invitrogen (Grand Island, NY). Teflon coated stainless steel annealed wire (0.010” bare, 0.013” coated) was purchased from A-M Systems, Inc. (Sequim, WA). A custom circuit stimulator model was designed and tested in our lab to generate a pulsed, direct current (DC) square waveform of 3 V, 20 Hz. The waveform enabled results to be directly comparable to prior work using a single electric field [51]. Circuit design was updated to include a magnetic switch for controlled activation/deactivation and prepared in quantity by Microcircuits Diversified, Inc. (Salisbury, NC).

### Synthesis and Crosslinking of the base polymers, PGS, and PGSA

PGS was prepared by melt synthesis of warmed glycerol dropwise added to sebacic acid (3:1 w/v sebacic acid:glycerol) followed by 4 h of 15 inHg vacuum pressure [80]. Molten PGS prepolymer was combined with excess dichloromethane (DCM) to enable formation of acrylated PGS (PGSA). Acryloyl chloride was added by % molar mass to achieve final acrylations of 25, 30, 35, and 40% acrylated PGSA. Triethylamine was added dropwise (1.2:1.0, triethylamine:acryloyl chloride) to force the solution from equilibrium and the resultant solution was stirred overnight. Residual triethylamine salts were removed by rotary evaporation of the DCM, precipitation of the salt in excess ethyl acetate, and filtration of the solution [79, 81]. Rotary evaporation was used to remove residual solvents by chemical displacement (DCM displaced ethyl acetate, ethanol displaced DCM).

To prepare PGS sheets, PGS was poured into a rectangular silicone mold and polymerized at ~150 °C under 51 kPa vacuum pressure until a glass transition temperature of approximately −25 °C was reached, as determined via differential scanning calorimetry (DSC). To prepare PGSA sheets, solutions of PGSA and 200 proof ethanol (1:3, ethanol: PGSA, by volume) were warmed (35–40 °C) to facilitate incorporation of 1% (w/w) 2,2-Dimethoxy-2-phenyl-acetophenone (DMPA), a photoinitiator. Following ethanol evaporation, PGSA was poured into parchment lined plastic molds and crosslinked via 20 min of UV exposure (365 nm, 30 mW/cm^2^, model 100AP, Blak-Ray (UVP, LLC., Upland, CA)). Using these processes, thin sheets (1.12 ± 0.032 mm (average thickness ± standard error of the mean)) of PGS and PGSA were prepared in triplicate.

### Synthesis of conductive dopant, (PPy-DEHS)

Chemically crosslinked PPy was synthesized in our lab from pyrrole doped with dioctyl sulfosuccinate sodium salt, C_20_H_37_NaO_7_S. Briefly, PPy was synthesized by dropping freshly distilled pyrrole into a chilled Na^+^DEHS^-^ solution (0.4 mol: 0.15 mol) with continuous stirring. A chilled solution of 0.10 mol of ammonium persulfate, (NH_4_)_2_S_2_O_8_ was dropped into the pyrrole/Na^+^DEHS^-^ and maintained at 4 °C for 20 h with continous vigorous stirring. The doped poly(pyrrole) precipitate was washed, centrifuged, filtered and dried prior to use [82, 83].

### Polymer characterization

Proton Nuclear Magnetic Resonance (^1^H NMR) was performed using a Bruker 300 MHz NMR equipped with 5 mm temperature regulated QNP probe (Bruker Biospin Corporation, Billerica, MA) to confirm consistency between polymer batches and to confirm the degree of acrylation [78]. Data were collected using TopSpin 1.3 and standard Bruker parameters at 25 °C. PGS and PGSA samples were dissolved in chloroform-d and assessed using MestReC v.4.9.9.5 NMR software (Mestrelab Research, Escondido, CA). Briefly, PGS and PGSA peaks differ by the presence of three acrylate peaks located at 5.9, 6.1, and 6.4 ppm. The calculated integrations for sebacic acid located at peaks 1.3 and 1.6 ppm were 8 and 4, respectively. The sum of the measured integrations for peaks at 1.3 and 1.6 ppm was divided by the calculated integration (12) to determine the relative ratio of sebacic acid. Acrylations performed at 25, 30, 35, and 40% were expected to yield three peaks at 5.9, 6.1, and 6.4 ppm of equivalent integration of approximately 0.25, 0.30, 0.35, and 0.40, respectively. The three vinyl peaks were integrated separately with calculated values of one each for 100% acrylation. Vinyl integrations were averaged and divided by the relative ratio of sebacic acid to determine the percent acrylation for each polymer.

A JASCO FT/IR 460 Plus Attenuated Total Reflectance—Fourier Transform Spectrometer (ATR-FTIR) (Oklahoma City, OK) was used to compare changes in bonds of PGS and the acrylated PGS molecules. UV-crosslinked polymer discs (2 mm diameter) were placed on the diamond crystal under contact pressure to eliminate the air/polymer interface. Crosslinked PGS was used as a baseline for the non-acrylated polymer bonds. Peaks for acrylation bonds were possible in the ranges 1125–1225, 1250–1325, and 1700–1750 cm^−1^.

X-ray Diffraction (XRD) collected angular scatter, which allows the atomic structure of molecules to be resolved. Diffraction data was collected at room temperature using a Bruker P4 general-purpose four-circle X-ray diffractometer modified with a GADDS/Hi-Star detector (Madison, WI) to monitor structural changes for heat polymerized PGS and UV polymerized PGSA with 25, 30, 35, and 40% acrylation. Samples of the polymers were cut into discs (2 mm diameter, 1.1 mm average thickness) using a punch biopsy. The goniometer was controlled using the GADDS software suite (Bruker, Madison, WI) with 4 min exposures in transmission mode. The system employed a graphite monochromator and a Cu Kα (λ = 1.54184 Å) fine-focus sealed tube operated at 1.2 kW power (40 kV, 30 mA). Data was reduced using area integration methods to produce diffraction patterns of intensity vs. 2θ for each sample and was analyzed with the program EVA (Bruker, Madison, WI).

Differential scanning calorimetry (DSC) was used to determine changes in polymer state with respect to changing temperature. A DSC Q 200 (TA Instruments (New Castle, DE)) assessed samples in Tzero pans and lids using a 3 cycle heat, cool, heat approach, namely (0 to 40 °C, 40 to −60 °C, −60 °C to 40 °C). Universal Analysis software (TA Instruments) was used to analyze output data for glass transition temperature (T_g_) based on the midpoint of inflection in the slope of the melt curves.

### Mechanical characterization and degradation

Prior to use with cells, materials must undergo cleaning to remove unreacted polymeric materials, followed by sterilization. To characterize the materials for biological application, analysis was performed on polymer samples which were untreated, as well as, treated for cell culture. Two different treatment methods were tested for the samples. Treatment 1 consisted of 3–1 h 200 proof ethanol washes followed by 3–1 h PBS washes [81]. An alternate treatment was developed following observation of microscopic fractures in the materials resultant from rapid swelling induced by the concentrated ethanol treatment. Treatment 2 was comprised of 15 min washes of gradated ethanol (30, 40, 50, 60, 50, and 30%) followed by triplicate, 15 min washes of PBS.

An Instron 300 R Mechanical Tester (Norwood, MA) was used to characterize the mechanical properties of the PGS and PGSA, respectively. Crosslinked samples were stamped into dog bone shapes (ASTM D-638-V) with dimensions 3.5 cm × 5 mm (length, width) and average thickness 1.1 mm. Tensile testing was performed at 50 mm/min with a 500 N load cell. Samples were prepared in triplicate with *n* = 9. Samples were evaluated for Young’s modulus, ultimate stress, ultimate strain. Young’s modulus was identified by the slope of the linear region of the stress-strain curve prior to break. Ultimate stress was calculated from the force at break per initial cross-sectional area. Ultimate strain was calculated by (elongation at break)/(initial length)*100%. Samples were compared per treatment type (dry, T1, or T2) with increasing acrylation and across treatment types (dry, T1, or T2) by pairing samples by acrylation.

Discs (~3 mm diameter, 1.1 mm average thickness) of PGSA with 25, 30, 35 or 40% acrylation were punched from sheets of polymerized material. The initial mass, diameter, and thickness of each sample was recorded for five samples from each group, as well as the initial PBS pH. Mass measurements were made using an XS64 analytical balance from Mettler Toledo (Columbus, OH). Diameter and thickness measurements were made with minimal contact measurements for each sample using Fowler Digital Calipers 54-100-000-2 with resolution of 0.01 mm and accuracy of 0.02 mm for MSC Industrial Supply (Melville, NY). Samples were separately placed in 5 mL of PBS. Diameter and thickness were measured daily for 7 days, then weekly until 140 days. After measurement, each sample was returned to fresh PBS and maintained at 37^o^C.

Separate samples were prepared for mass loss with five samples per group for each time point (daily for 7 days, then weekly for 140 days). The dry mass of each sample was recorded and the mass loss determined by the equation: $\% {\rm{loss}}=\frac{{{\rm{M}}}_{{\rm{i}}}-{{\rm{M}}}_{{\rm{f}}}}{{{\rm{M}}}_{{\rm{i}}}}100$, where M_i_ is the initial mass and M_f_ is the final, dry mass each week as measured following lyophilization for 7 h.

### Biocompatibility

For biocompatibility, 2 × 2 cm square samples were prepared for cell culture using treatment two. Six well tissue culture polystyrene plates were prepared with either no sample (control), a disc sample of 35% acrylation, and a disc sample of 40% acrylation per well. Samples were evaluated using both HEPM (fibroblast morphology) and B35 (neuronal morphology) cell lines. All samples were seeded with 300,000 cells independent of cell type and maintained at 37 °C, 5% CO_2_ in DMEM with high glucose for three days. Three additional samples were prepared with 300,000 cells each using lysis buffer to get a baseline of initial cell protein prior to division. Media was changed daily and cells were lysed after three days. All samples were prepared in triplicate using the standard bicinchoninic acid (BCA) assay to quantify the protein mass from each well.

### Electrical conductivity

Polymeric samples for electrical testing were prepared as discs (~9.5 cm Ø, 1.3 mm average thickness). To produce discs, solutions of 0, 0.001, 0.005, 0.05 % (wt/wt) of PPy in PGSA, ethanol based PPy solutions (bath (1 h) and horn sonicated (30% power, 2 s on/off, 1 h)) were added to PGSA/ DMPA/ethanol solutions. Combined solutions were bath (1 h) and horn sonicated (30% power, 2 s on/off, 1 h) and poured into parchment paper lined dishes for overnight solvent evaporation followed by 20 min of UV crosslinking. Disc volume resistance (R) was measured using a Keithley Model 65 High Resistivity Test System (Cleveland, OH) (V range = 10 V, i = 100 nA, 500 readings per sample) and sample thickness (t) using digital calipers to obtain R_avg_ and t_avg_, respectively. Conductivity was calculated using t_avg_/R_avg_.

### Biphasic sheet preparation

**Fig. 2:**
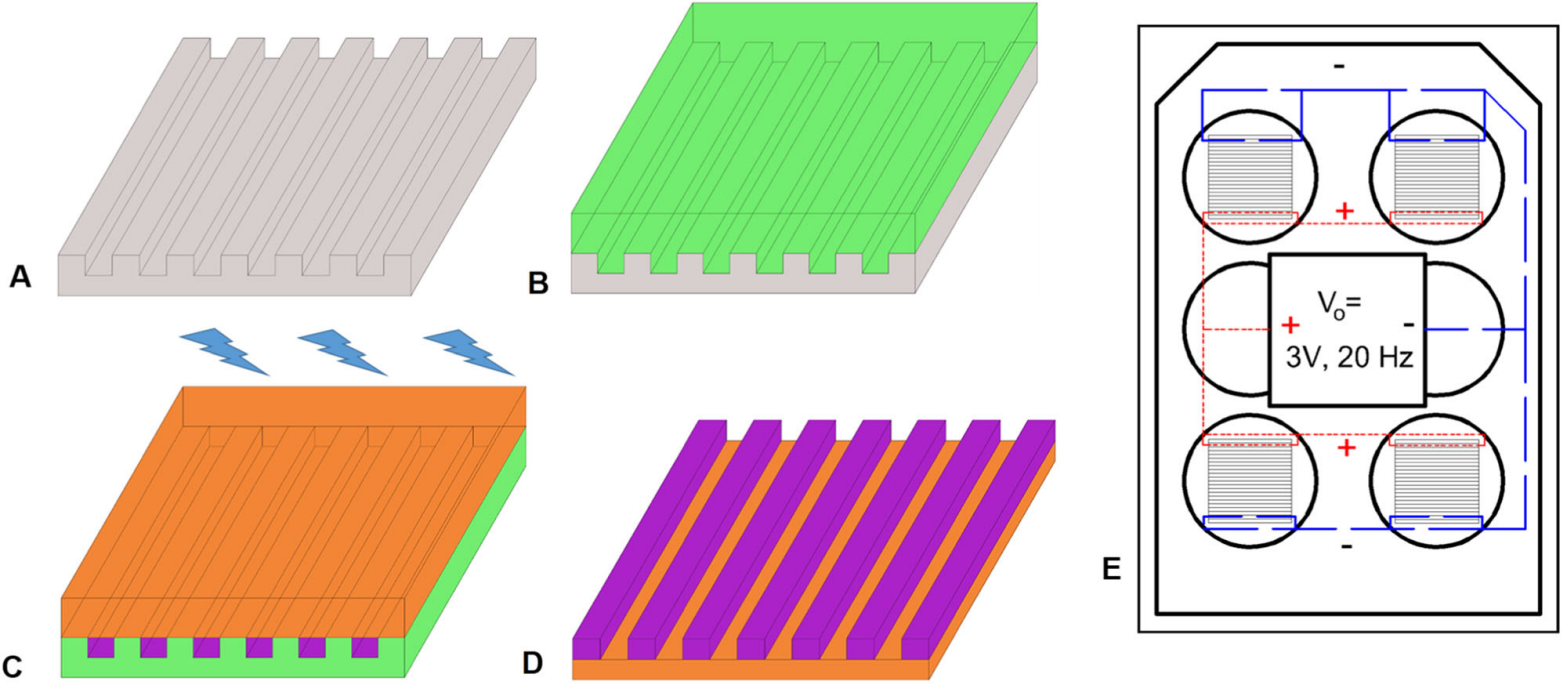
Preparation of Biphasic Sheet for In Vitro Evaluation with 2A-2D from a zoomed in perspective **A** An inverse mold with spacings 600 μm wide was used to **B** cast a silicone mold. **C** Using the flexible silicone mold, low conductivity polymer was injected into the mold valleys and high conductivity polymer covered the surface followed by UV crosslinking for 20 min at 365 nm, 30 mW/cm^2^ **D** Materials were peeled off the silicone mold to obtain a biphasic conductive sheet. **E** Biphasic sheets were placed in a modified six well plate for cell stimulation. Long dashes represent wires connected to the circuit ground and dotted lines represent wires connected to the positive circuit output

The goal of this study was to investigate a sheet of materials which could later be reproduced as a conduit capable of producing repeated electric field gradients for peripheral nerve repair. This goal limited strategies for construction techniques due to feature size (600 μm segments) and limits of injection and extrusion molding. To achieve this alternating material structure and keep the conductive elements connected across the length of the sheet (and prospective tube), a two step mold process was used, which enabled injection of the low conductivity sections into the mold slits, followed by a continuous layer of the high conductivity material on top. This resulted in a shallow, ribbed structure capable of either flat or conduit formation. Further, prior topographical studies have shown that micro and nanostructures have not impeded continued axonal growth [84–86]. Figure 2 illustrates a zoomed in perspective of the molding process.

Preparation of a sheet mold began with a Delrin inverse mold with spacings 600 μm wide and 400 μm deep (Fig. 2A). Using Sylgard 184 Silicone Elastomer Kit, silicone molds were cast using the Delrin mold to obtain thin flexible sheets with the desired spacings which could be used to create tubular molds in the future (Fig. 2B). Low conductivity polymer was injected into the valleys of the mold and the top was coated with high conductivity polymer (Fig. 2C). Sheets were crosslinked for 20 min under 365 nm, 30 mW/cm^2^ UV irradiation (Fig. 2C) to obtain a final biphasic sheet (Fig. 2D).

### In vitro stimulation

A six-well plate was modified to incorporate wires for stimulation connected to a central circuit capable of providing a 3 V, 20 Hz square-wave pulse to connected sheets. Wires were bent in a square wave formation and fixed in position to the top of the wells using hot glue on regions of insulated wire in parallel across each corner well. The “square” wave shape of the wired allowed the uncoated wires within the well to be adjusted within slits of the treated sheet to stimulate and hold the sheet in position. Following construction, the modified six well plates were rinsed with ethanol and sterilized with ethylene oxide (EtO) gas. In a sterile tissue culture hood, an EtO sterilized, treated sheet was placed in each well, then held in place by the wire leads used for electrical stimulation (See Fig. 2E). To approximate axonal response to the conducting biphasic sheets, B35 (neuronal morphology) cells were seeded at 300,000 per well onto tissue culture polystyrene with no sample (control) or biphasic material. After 12 h of attachment, circuits were activated for 0, 1, or 3 days and cells were imaged at 12 h intervals for 3 days. Samples were imaged using a timelapse Axiovert 100 inverted microscope at 32x magnification using Open Lab software (Waltham, Massachusetts) with climate control chamber (37 °C, 5% CO_2_). The biphasic nature of the materials resulted in hills of low conductivity regions and valleys of high conductivity. The plane of focus was set to cells in the high conductivity region with a clear region of low conductivity located on one side of each image. Cells were counted based on region occupied (low/high conductivity border, 0–200 μm from the low/high conductivity border, and 200–400 μm from the low/high conductivity border), which indicated cell proliferation and spreading during the division process across the region’s electric field.

### In vivo evaluation

**Fig. 3:**
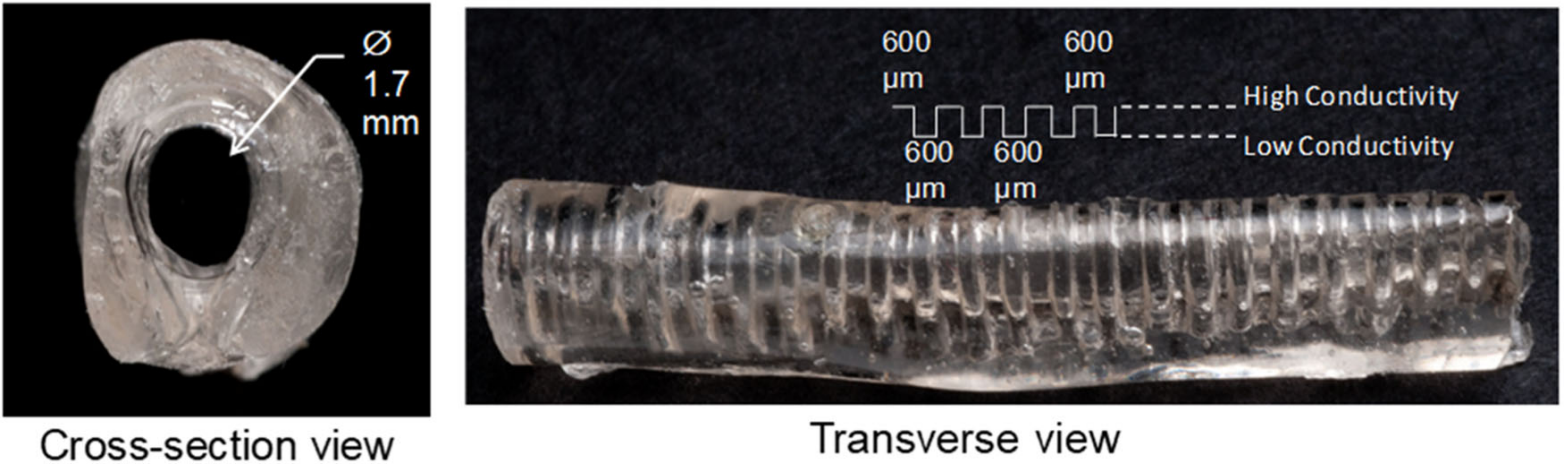
Cross-sectional and transverse views of the biphasic conducting tube for implantation in sciatic nerve injury model showing the alternating high and low conductivity regions

For preliminary evaluation of the Biphasic material in vivo, a tube was created to serve as a nerve conduit, similar to the conduits used in clinical management of peripheral nerve injuries. An inverse mold with 600 μm wide and 400 μm deep spacings was first produced using Sylgard 184 Silicone Elastomer Kit (Dow Corning, Midland, MD). Silicone templates were secured around a 30 G wire to form a tube. Silicone molds were filled with 7% sodium alginate in PBS and placed into a 0.5 M CaCl solution to crosslink the alginate gels. Then the templates were injected with low conductivity polymer solution and the tube exterior coated with high conductivity polymer. This produced tubes with an outside diameter of 4.0 ± 0.2 mm; inside diameter of 3.0 ± 0.3 mm, and 0.5 mm wall thickness, as shown in Fig. 3.

Male Lewis rats were used for histological evaluation in a transected sciatic nerve injury model. Animal studies were approved by the Institutional Animal Care and Use Committee (IACUC) at Wake Forest University School of Medicine. Rats were anesthetized via inhalation isoflurane throughout surgery, and were randomly selected for treatment with a silicone conduit, or a biphasic conducting conduit. The right hind leg was aseptically prepared, and an incision was made through the skin for retraction of the biceps femoris and gluteus maximus muscles to expose the sciatic nerve. A one cm gap in the nerve was created, starting 3 mm below the tendon at the hip. Each tube was sutured with 10/0 suture at the proximal and distal ends of the sciatic nerve. Muscle tissue and skin were then closed with 4/0 prolene. Following surgical recovery rats were unable to move their right lower limb. Analgesia was provided via subcutaneous injection of buprenorphine (0.01 mg/kg). Two weeks post-surgery animals were euthanized using heavy isoflurane anesthetization until breathing discontinued, followed by decapitation. Sciatic nerves with implants were harvested and placed into chilled (4 °C) 4% neutral buffered formalin for 48 h, then rinsed with PBS, cross-sectioned into thirds, and separated by location (proximal, middle and distal). Samples were post-fixed in 2% osmium tetroxide, then rinsed in PBS, and dehydrated in a gradated series of ethanol, followed by propylene oxide, and infiltrated overnight in 1:1 propylene oxide and spur resin. Tissue Section (1 μm) were mounted onto slides and stained with toluidine blue, and imaged using a 100x objective on a Zeiss light microscope (Zeiss Microscopy, Jena, Germany).

### Statistical analysis

All quantitative data were shown as mean ± standard deviation with experiments performed in triplicate. Mechanical testing resulted in uneven n’s due to some samples breaking in the grips. Statistical analysis for mechanical testing was performed using Wilcoxon’s signed rank test via SAS with *p* < 0.01. All other statistical analysis of samples was performed via ANOVA with Holm–Sidak for post hoc evaluation of significant differences among groups. Statistical significance was determined by *p* < 0.05, *α* < 0.01.

## Results

### ^1^H NMR characterization of PGS and PGSA

**Fig. 4:**
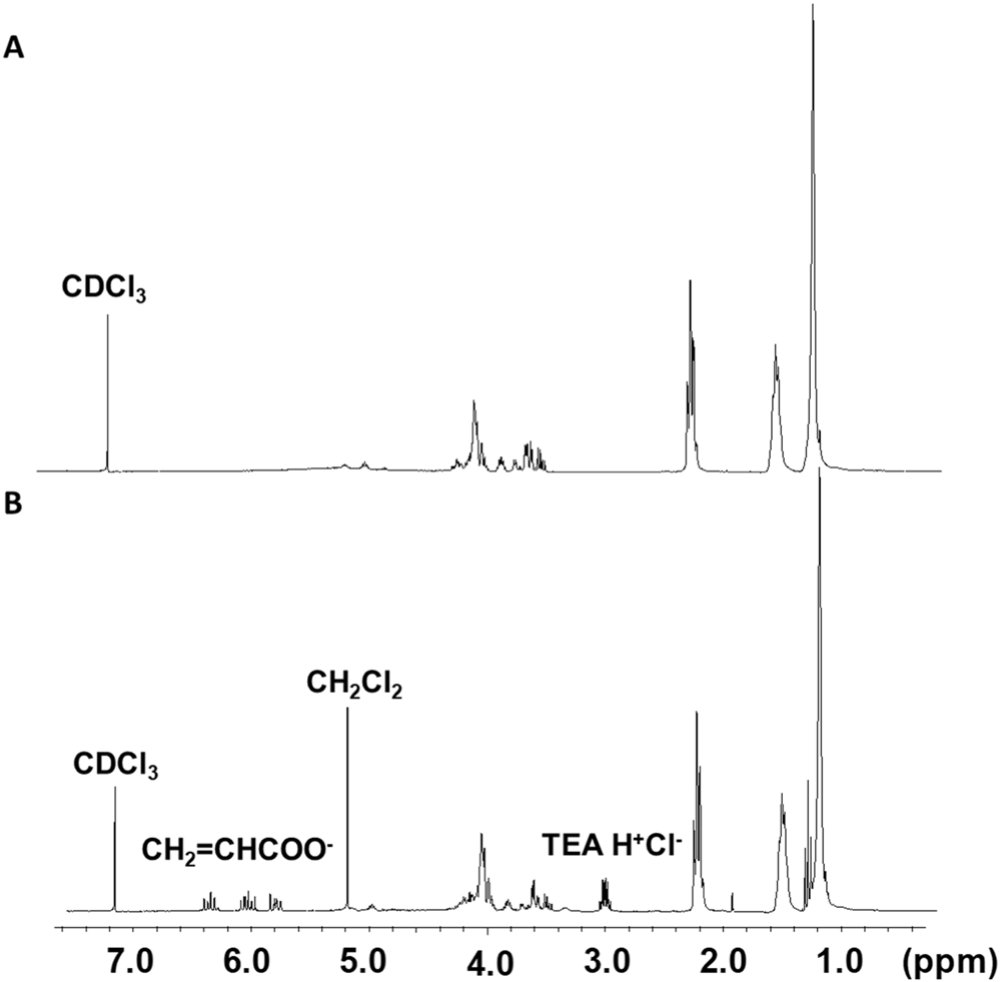
^**1**^H NMR of prepolymers: **A** PGS. Sebacic acid peaks were observed at 1.2,1.5, and 2.2 ppm. Glycerol peaks were at 3.7, 4.2, and 5.2 ppm. **B** PGSA 40% acrylation, vinyl peaks were observed at 5.9, 6.1, and 6.4 ppm, in addition to sebacic acid and glycerol peaks as observed in PGS prepolymer. Residual dichloromethane (CH_2_Cl_2_) and triethylamine hydrochloride (TEA H^+^Cl^-^) were observed at 5.3 and 3.0 ppm, respectively

PGS was assessed via ^**1**^H NMR to confirm consistency with previously reported ^**1**^H NMR (Fig. 4A) [78–81]. PGSA prepared from acrylated PGS prepolymer was assessed via ^**1**^H NMR and compared to PGS prepolymer results, as well as, previously reported ^**1**^H NMR (Fig. 4B) [78, 79, 81]. PGS prepolymer was consistent with previously reported 1:1 sebacic acid to glycerol ratio. PGSA acrylation was designed to produce 25, 30, 35, and 40% acrylated prepolymer. Final calculated acrylations were then 24.1, 27.7, 36.2, and 39.4% acrylated PGSA. These values are reasonable with consideration to ^**1**^H NMR error in integration being up to 10%. With error in mind, these values can be reasonably referred to as 25, 30, 35, and 40% acrylated PGSA. Consistency with batches was verified using ^**1**^H NMR for all prepared PGSA.

### Structural characterization of crosslinked PGS and PGSA

**Fig. 5:**
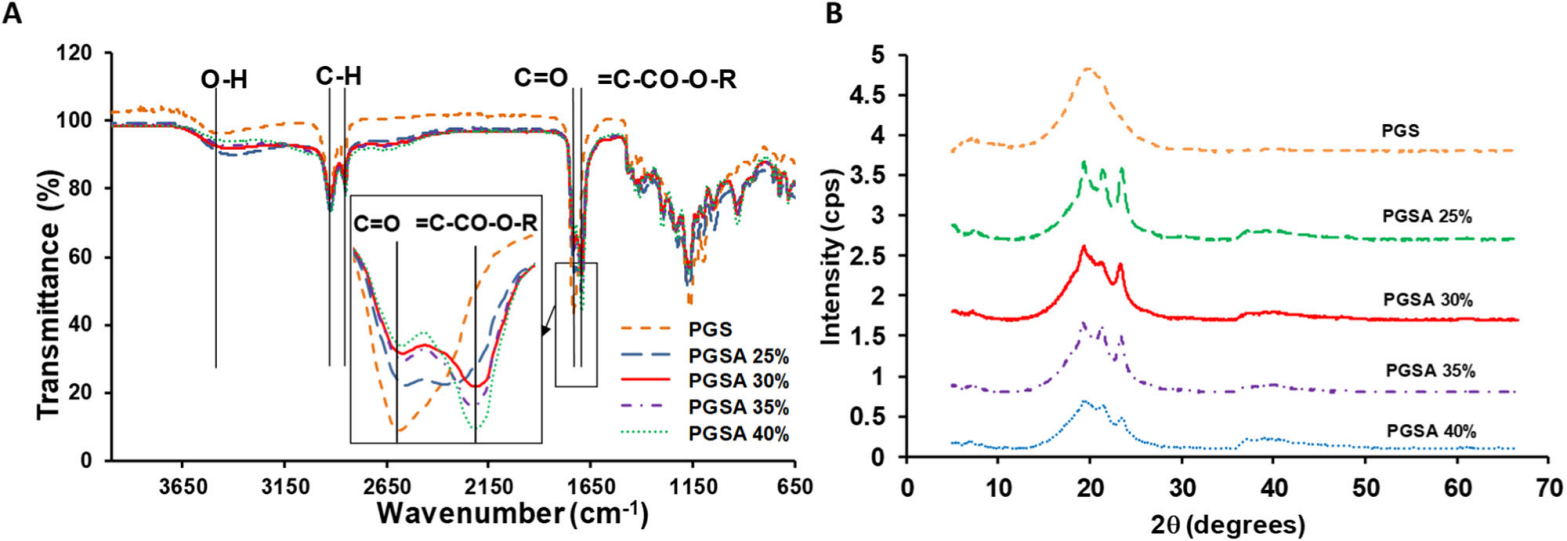
**A** ATR-FTIR spectra of untreated PGS and PGSA with 25, 30, 35, and 40% acrylation. Spectra for PGSA were the same as PGS with the addition of an acrylate stretch at 1693 cm^−1^. Carbonyl bonds are present in all polymers but decrease in intensity with increasing acrylation. **B** Fourier transformed X-ray diffraction patterns for PGS and PGSA with 25, 30, 35, and 40% acrylation treated to remove unreacted agents. Specimens were free standing films of 0.6 mm thickness. Characteristic peaks are observed at 7.2, 19.9, 31.5, 37.2, and 45.5° along 2*θ*

ATR-FTIR was used to confirm incorporation of acrylation of PGS by comparison of UV-crosslinked PGSA and thermally cured PGS (Fig. 5A). For PGS, the C = O bond was observed at 1700 cm^−1^, the stretch observed from 2850 to 2920 cm^−1^ was attributed to C-H, and the O-H stretch was observed around 3300 cm^−1^ (Fig. 5A, B). For PGSA, the C = C bonds of the acrylate have typical resonations at 1125–1225, 1250–1325, and 1700–1750 cm^−1^. The spectra for PGSA were the same as PGS with the addition of an acrylate stretch at 1693 cm^−1^ (Fig. 5A).

XRD was used to evaluate structural changes of the PGS post acrylation by comparing thermally cured PGS and UV-cured PGSA. Thermally cured PGS has a characteristic broad “amorphous” peak centered at 2θ ~ 20° and a minor peak at 7.2° (Fig. 5B, Table 2). PGSA has characteristic peaks at 19.35, 21.3, and 23.45° and a minor peak at 37.2°. At 7.2°, the peak intensity drops by 30% for PGSA 25% and 80% for PGSA 30, 35, and 40% acrylation compared to PGS. The amorphous peak of PGS appears to sharpen into 3 semi-crystalline peaks. The peak intensities generally decreased as acrylation increased for all PGSA peaks other than 7.2° and PGSA 40% at 37.15° (Fig. 5B, Table 2).

**Table 2:** X-ray diffraction characterization for 0–40% acrylation indicates intensity peaks occur at $2\theta$ = 7.2, 19.9, 21.3, 23.35 and 37.2°. Intensity and d-spacing are shown for each $2\theta$ peak

	PGS	PGSA 25%	PGSA 30%	PGSA 35%	PGSA 40%
2θ peak (°)	7.2	7.15	7.35	7.2	7.2
max intensity	0.161	0.1136	0.0324	0.0327	0.0335
d (nm)	12.27	12.35	12.02	12.27	12.27
2θ peak (°)	19.9	19.3	19.35	19.3	19.4
max intensity	1.025	0.9651	0.9175	0.8565	0.6001
d (nm)	4.46	4.60	4.58	4.60	4.57
2θ peak (°)		21.35	21.25	21.3	21.5
max intensity		0.8608	0.7195	0.8235	0.5419
d (nm)		4.16	4.18	4.17	4.13
2θ peak (°)		23.45	23.3	23.35	23.45
max intensity		0.8818	0.7042	0.7024	0.3831
d (nm)		3.79	3.81	3.81	3.79
2θ peak (°)		37.1	37.3	37.2	37.15
max intensity		0.1102	0.0879	0.079	0.1204
d (nm)		2.42	2.41	2.41	2.42

**Fig. 6:**
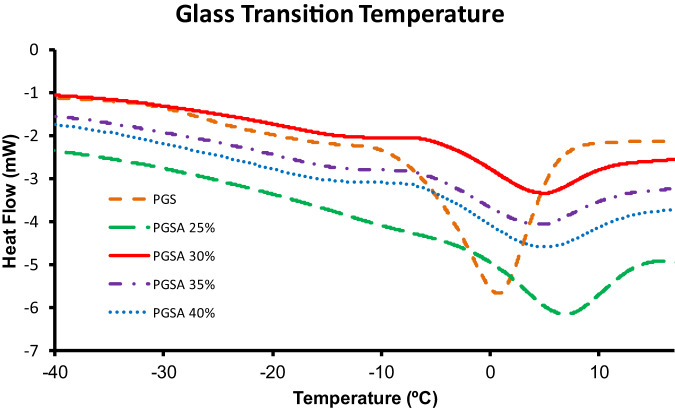
DSC glass transition and melt curves

DSC results showed PGS had a T_g_ of −25.7 ± 0.32 °C (mean ± SEM) after crosslinking at 150 °C (Table 3). PGSA demonstrated decreasing T_g_ as acrylation increased (e.g., T_g_ : −20.1 ± 0.15, −23.2 ± 0.43, −25.4 ± 0.30, −26.24 ± 0.43 °C (mean ± SEM): 25, 30, 35, 40% PGSA) (Table 3, Fig. 6). Significant differences were observed between 25%, 30%, and 0, 35, and 40% acrylations (*p* < 0.0001).

**Table 3:** DSC glass transition temperatures (T_g_) for PGS and PGSA with 25, 30, 35, and 40% acrylation.

	Tg (°C)
PGS	−25.68 ± 0.33
PGSA 25%	−20.06 ± 0.15
PGSA 30%	−23.16 ± 0.43
PGSA 35%	−25.41 ± 0.3
PGSA 40%	−26.24 ± 0.43

All values represent the mean ± standard error of the mean. No significant difference was observed between 0, 35, and 40% acrylations. Significant differences were observed between 25%, 30%, and 0, 35, and 40% acrylations (*p* < 0.0001)

### Mechanical characterization of crosslinked PGS and PGSA

**Fig. 7:**
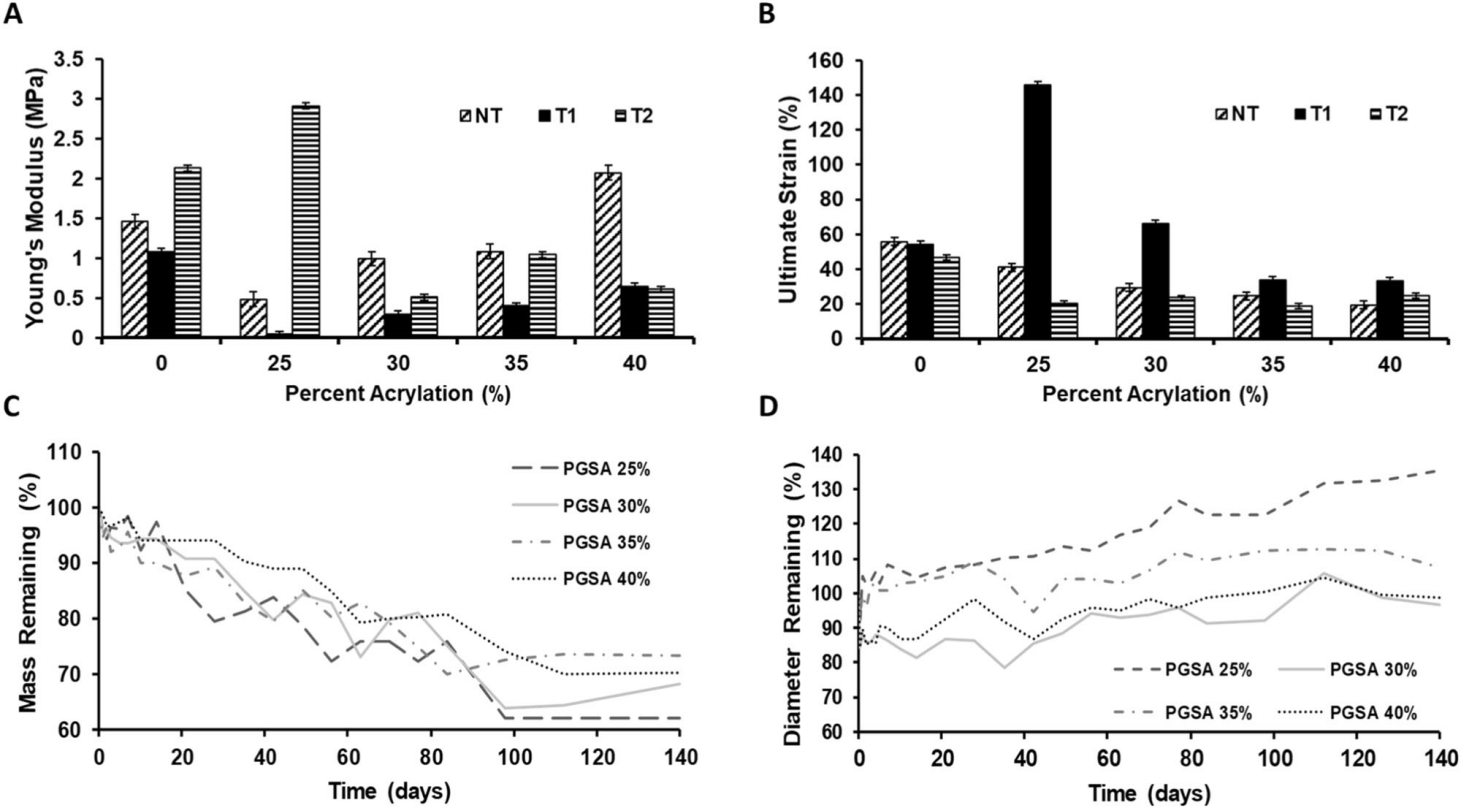
**A** Elastic moduli comparison of untreated PGS and PGSA with 25, 30, 35, and 40% acrylation. **B** Percent strain comparison of PGS and PGSA 25, 30, 35, and 40% acrylation prior to and following sample treatment for cell culture. The legend keys represent NT: untreated, T1: treatement 1 (3x repeated 70% ethanol washes), T2: treatment 2 (grated ethanol washes). Data presented as mean ± standard error of the mean. **C** Degradation based on Mass Remaining indicates that 70% of the mass remains after 20 weeks and that there is no difference in mass loss between different degrees of acrylation of PGSA. **D** Degradation based on swelling of the polymer demonstrates that all acrylations initially decrease in diameter followed by gradual swelling. For 25% acrylation, the swelling was more rapid with a final increase of 30% in diameter by 20 weeks; whereas for higher acrylations, swelling returned the discs to their original diameter by 20 weeks

Tensile testing was performed to assess the mechanical properties of thermally crosslinked PGS and UV-crosslinked PGSA with 25, 30, 35, and 40% acrylation. Samples were assessed after no treatment, treatment 1 (T1) or treatment 2 (T2) (Fig. 7A). The Young’s moduli of untreated samples exhibited a significant decrease (*p* < 0.0001) from PGS to PGSA 25%; however, the moduli increased significantly with increasing acrylation (*p* < 0.0001), such that, the modulus of 40% PGSA was equivalent to PGS (*p* > 0.01). There was also no significant difference (*p* > 0.01) in elastic modulus between untreated PGSA 30 and 35%. Samples which underwent T1 demonstrated the same trend as untreated samples with moduli values significantly lower (2–4x, *p* < 0.001) than that of untreated samples. The moduli for T1 treated PGSA samples increased with acrylation (*p* < 0.001) but all of them were significantly lower (*p* < 0.001) than T1 treated PGS. T2 treated PGSA 25% samples were significantly stiffer (6x, *p* < 0.001) than untreated. PGSA samples which underwent T2 exhibited a trend of generally decreasing moduli as acrylation increased. The modulus for PGSA 30% after T2 was atypically low for the trend but repetition confirmed the data were consistent (Fig. 7A). Moduli for T1 and untreated PGS and PGSA were significantly different (*p* < 0.001) for all pairs. Moduli for T2 and untreated PGS and PGSA samples were significantly different (*p* < 0.001) for all pairs except PGSA 35% (*p* > 0.01). Moduli for PGS and PGSA samples compared for treatments T1 vs T2 but all were significantly different (*p* < 0.0001) except for PGSA 40% (*p* > 0.01) (Fig. 7A). The modulus data demonstrate that PGS is stiffer than low acrylation PGSA but that moduli increased with increasing acrylation. It is evident that treatment for cell culture changes the mechanical properties of PGS and PGSA with T1 resulting in significantly more elastic samples than T2. T2 samples are more similar to untreated than T1, which suggested that T2 may be less harsh and more protective of the mechanical structure.

Examination of ultimate strain for untreated PGS and PGSA samples indicated a consistent significant decrease in strain (*p* < 0.0001) with increasing percent acrylation (Fig. 7B). Samples subject to T1 demonstrated a significant increase (3.5x, *p* < 0.0001) in strain from PGS to PGSA 25%, after which strain decreased significantly (*p* < 0.0001) with increasing percent acrylation. PGS and PGSA samples subject to T2 showed a significant decrease in strain (3.7x, *p* < 0.0001) from PGS to PGSA 25%; however, there was no significant change (*p* > 0.01) in strain with further increased in acrylation. Comparison of samples by acrylation for untreated vs T1 showed no significant difference (*p* > 0.01) for PGS, and significantly higher strain (*p* < 0.0001) for all acrylated samples. In contrast, untreated vs. T2 treated samples’ strain were only significantly different (*p* < 0.001) for PGSA 25%. Comparison of T1 vs T2 treatments demonstrated no significant differences (*p* < 0.0001) for PGS and PGSA 40%. However, T1 vs T2 comparisons for PGSA 25, 30, and 35% were significantly different (*p* < 0.0001) (Fig. 7B). The ultimate strain data indicate elongation decreases from PGS to PGSA 25% and decreases with increasing acrylation. T1 PGSA samples underwent significantly more strain than T2 and untreated samples with T2 maintaining similar values to that of untreated samples. This further indicates that T2 treatment may preserve the mechanical integrity of the PGSA polymers.

**Fig. 8:**
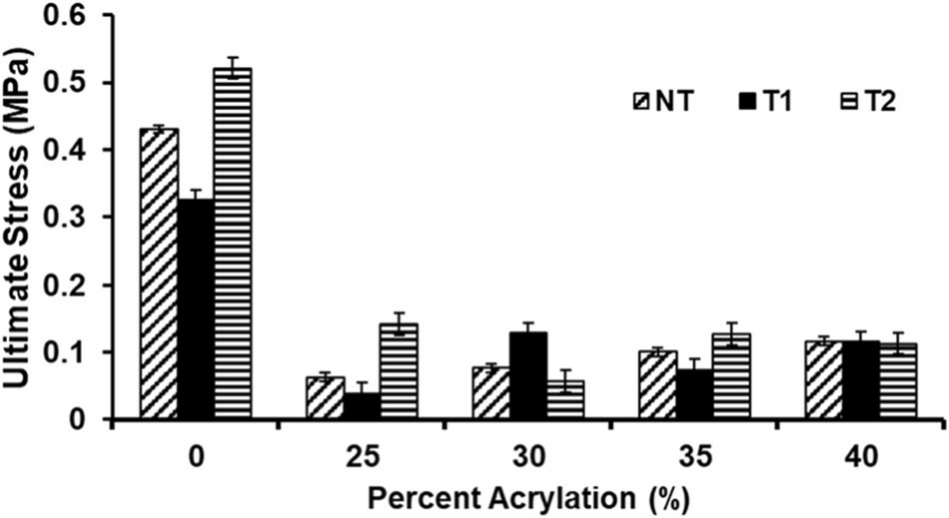
Ultimate stress comparison of untreated PGS and PGSA with 25, 30, 35, and 40% acrylation; where NT untreated, T1 treatment 1 (repeated 70% ethanol washes), T2 treatment 2 (gradated ethanol washes). Data presented as mean ± standard error of the mean

Untreated samples demonstrated a sharp decrease (*p* < 0.0001) in ultimate stress (stress at max load) from PGS to PGSA, and significant increases (*p* < 0.001) in stress with increasing PGSA. Stresses for all untreated PGSA samples were significantly different (*p* < 0.001) except for PGSA 35% vs 40% (*p* > 0.01) (Fig. 8). Samples which underwent T1 exhibited the trend of PGS being significantly greater than PGSA samples (*p* < 0.001). T1 PGSA samples showed no signifcant difference (*p* > 0.01) between 30%, 35%, and 40% but 25% sustained significantly less stress (*p* < 0.001) at max load compared to the other T1 treated PGSA samples. PGS samples were strongest in the T2 treated group with a stress at maximum load of 0.52 MPa. T2 samples’ strengths dropped sharply from PGS to PGSA 25 (*p* < 0.0001) and followed a trend of similar strength with no significant difference (*p* > 0.01) in stress at max load with increased acrylation, with exception to 30% acrylation which was significantly greater (*p* < 0.001) (Fig. 8). Comparison of sample stresses for untreated vs. T1 by acrylation percent demonstrated that the stress withstood by untreated PGS and PGSA 25 were significantly greater (*p* < 0.0001) than T1 treated PGS and PGSA 25%. In general, as the percent acrylation increased the untreated and T1 samples approached more similar stresses at maximum load with no significant difference (*p* > 0.01) between the PGSA 40% for untreated vs T1 samples, all other pairings were significantly different (*p* < 0.0001). Comparison of untreated vs. T2 samples was reversed. Ultimate stresses for T2 treated samples were generally statistically higher than that of untreated with all samples being significantly different (*p* < 0.0001) except PGSA 40% (*p* > 0.01). T1 vs T2 comparison by acrylation demonstrated that T2 generally withstood significantly higher stress (*p* < 0.0001) than samples exposed to T1, with exceptions of PGSA 30% (which was significantly lower, *p* < 0.0001) and PGSA 40%, which were not significantly different (*p* > 0.01) (Fig. 8). Ultimate stresses for PGS samples were significantly higher than the PGSA samples. Untreated and T1 PGSA increased with increasing acrylation but T2 PGSA 25, 35, and 40% were not significantly different.

Taken together, the mechanical testing data indicate that treatment 2 (T2) appears to preserve mechanical properties of PGSA polymers. Modulus and ultimate strain are met by T2 samples with the noted exception of irregularity of PGSA 30%. The data were duplicated independently and the results remained unchanged. For completeness, degradation data were continued with T2 treated PGSA samples. The mechanical testing data served to reduce samples for subsequent cell culture studies. Based on consistency and meeting the target nerve measures shown in Table 1, cell studies were continued with PGSA 35% and 40% with their key difference being stiffness, a factor which can influence cell response.

Degradation properties were evaluated for all PGSA samples. There was no significant difference in degradation between the different acrylations for mass remaining. Mass remaining dropped to 96% over the first 24 h, and then decreased linearly at 0.32% per day for 14 weeks. After 14 weeks, the remaining mass stabilized at 70% throughout the rest of the 20 weeks (Fig. 7C). The swelling data only demonstrated a significant difference between 25% and the higher acrylations. During the first 24 h, there was an average of a 12% decrease in diameter across samples. For the higher acrylations, swelling increased at 0.18% per day up to 11 weeks to stabilize at ~100% for the remainder of the 20 weeks (Fig. 7D).

### Cellular compatibility

**Fig. 9:**
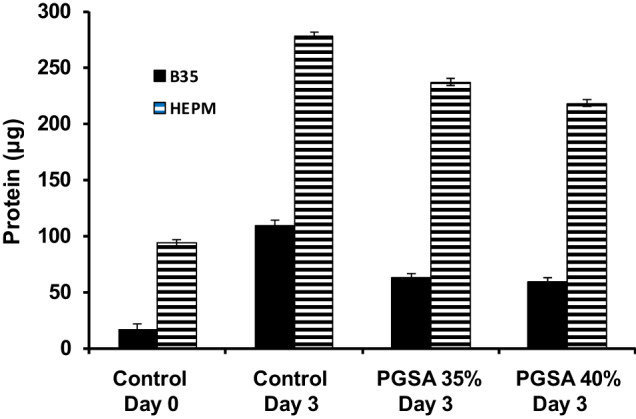
BCA Protein Assay comparison of HEPM (fibroblast morphology) and B35 (neuroblastoma morphology) cells prior to plating (nonplated), and cells plated on tissue culture polystyrene, PGSA 35% acrylation, and PGSA 40% acrylation for three days growth. Data presented as mean ± standard error of the mean. There was no significant difference between 35 and 40% acrylation (*p* > 0.05)

BCA protein assays were performed using HEPM and B35 cell lines. For HEPM cell lines, there was no significant difference between 35 and 40% PGSA. There were significant differences between the non-plated cells (i.e., cell samples collected at same time as seeding to establish initial protein values) and the cells seeded onto control and biphasic polymer sheets. From day 0 to day 3, cells proliferated on all materials with increases of 1.96x, 1.52x, and 1.32x for control, 35% PGSA, and 40% PGSA, respectively (Fig. 9). For B35 cells, cells proliferated from non-plated to plated conditions with increases of 5.6x, 2.78x, and 2.58x for control, 35%, and 40% PGSA. There was no significant difference between 35% and 40% PGSA (Fig. 9).

### Electrical conductivity

**Fig. 10:**
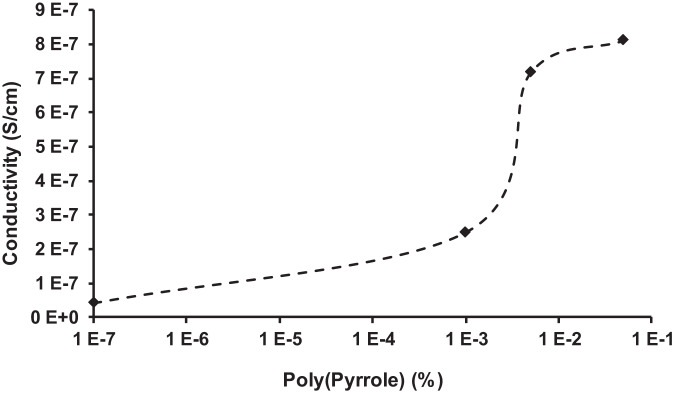
Conductivity comparison of PGSA with 40% acrylation doped with 0.001, 0.005, and 0.05% PPy. PGSA alone had a conductivity of 3 × 10^−8^ S/cm. PGSA conductivity was enhanced by incorporating PPy. The percolation threshold was reached at 0.0025%. The percolation limit was approached at 0.05% PPy (~9 × 10^−7^ S/cm)

Electrical conductivity data were performed on PGSA doped with 0, 0.001, 0.005, and 0.05 % soluble PPy. There was a significant difference between all samples, *p* < 0.001, *α* = 0.05. PGSA alone demonstrated low level conductivity of 1E-8 S/cm. The percolation threshold was at ~0.0025% (~5E-7 S/cm) and the percolation limit was found at 0.05% (~1E-6S/cm) (Fig. 10).

### In vitro cellular stimulation via biphasic sheets

**Fig. 11:**
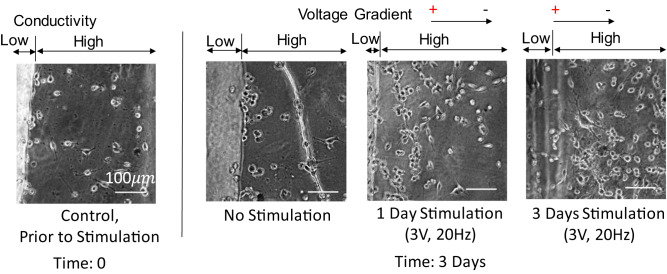
Cellular replication, migration, and direction as influenced by biphasic sheets alone or with 1 or 3 days of electrical stimulation at 3 V, 20 Hz. After three days of cell culture, images indicated 3 days stimulation >1 day stimulation > no stimulation, in terms of cell migration across the high conductivity regions and cell number. Images were captured at 32x magnification

Cellular growth was compared over time on the biphasic polymer sheets with either 0, 1, or 3 days of stimulation at 3 V, 20 Hz through the biphasic sheets (Fig. 11). The image shown at time 0 is representative of that observed prior to stimulation on the materials with cells evenly distributed across the high conductivity region.

After three days, control sheets (no stimulation) showed a higher proportion of cells adhering to the border between the high and low conductivity materials with axons connections parallel to topographical features and fewer cells across the high conductivity area than at 12 h adhesion. There are cells just out of focus on the low conductivity region but they lack apparent axonal extensions (Fig. 11).

After 3 days, sheets which had no stimulation had doubled along the border of low/high conductivity but have the same number of cells from the 100–400 μm from the border of low/high conductivity. Of note, the cells have noticeably long connections in the 100–400 μm range from the low/high conductivity border for sheets without stimulation and out of focus cells in the low conductivity region lack apparent axonal extensions (Fig. 11).

After three days, sheets with 1 day of stimulation in vitro demonstrated a 5x increase in cells from the low/high conductivity border up to 200 μm away in the high conductivity region and a 2x increase in cells from 200–400 μm from the low/high conductivity border. Further, there were cells with axonal extensions crossing the borders between high and low conductivity, which were apparent as they shifted out of the focus plane (Fig. 11).

At day 3, sheets with 3 days of stimulation in vitro indicate that the cells have proliferated further to have 2x the cells from day 0 at the border, 10x the cells within 200 μm of the border between high and low conductivity, and 7x the cells in the middle of the high conductivity region (~200–400 μm from the high/low conductivity border) (Fig. 11). In the low conductivity plane, cells with axonal extensions are apparent. These data indicate both significant migration and proliferation of the cells from the borders and directed across the gap regions due to electrical stimulation.

### In vivo evaluation of the biphasic conduits

**Fig. 12:**
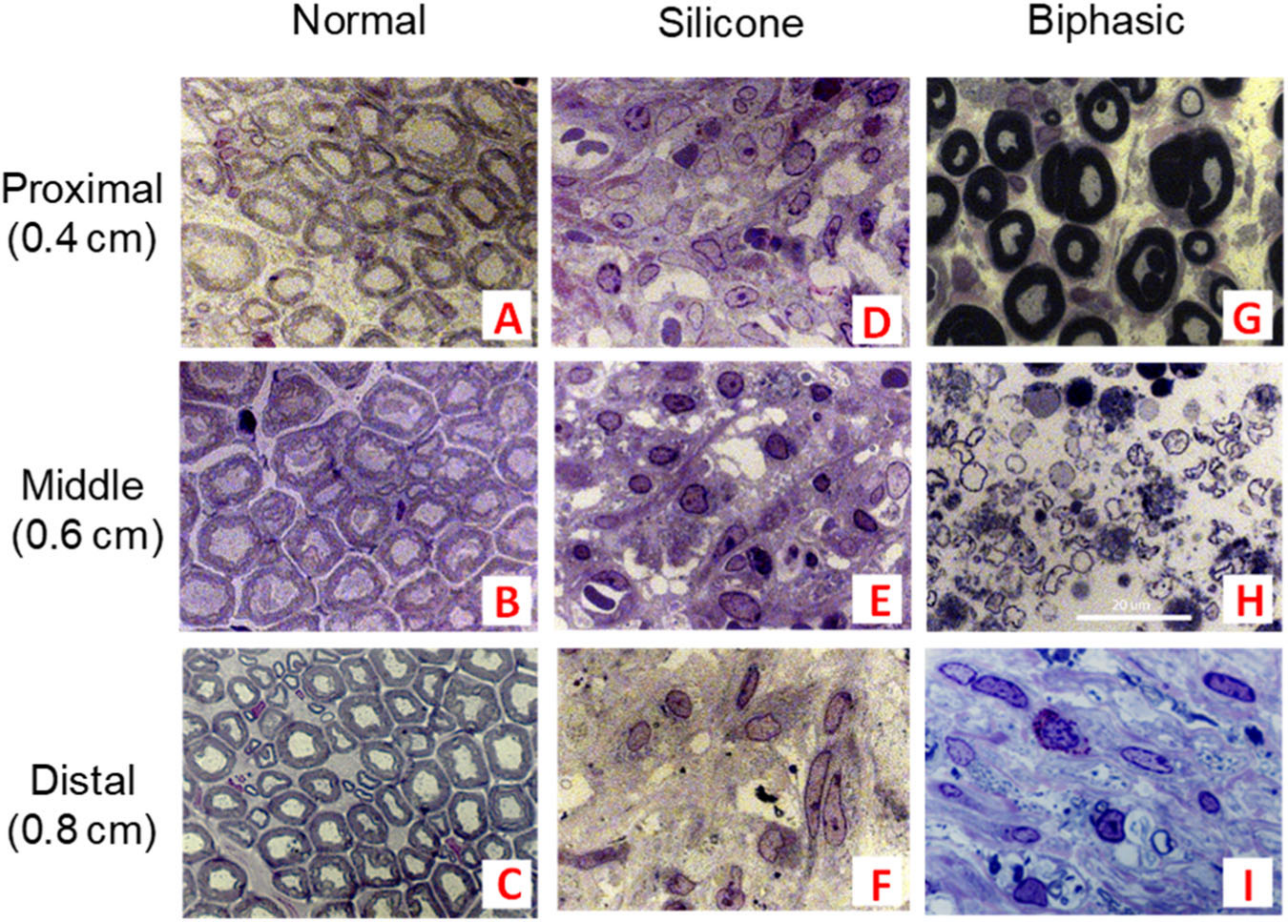
Cross-sections of sciatic nerves from proximal to the middle of the injury (0.4 cm), mid injury (0.6 cm), and distal to the middle of the injury (0.8 cm). Excised nerves groups are indicated by **A**–**C** uninjured normal nerve, **D**–**F** silicone tube, and **G**–**I** biphasic tube

Images of sciatic nerves from the middle, proximal, and distal portions of the conduit are shown in Fig. 12. Un-injured nerves exhibit heavily myelinated axons with close-packed axonal structures few unmyelinated axons. Silicone tubes exhibit predominantly unmyelinated axons, and evidence of fibrous tissue in the proximal and middle regions, and have fewer axons in the distal portion. Nerves treated with biphasic tubes had axons with good myelination in the proximal region, and minimal fibrosis throughout.

## Discussion

### Validation of PGSA materials properties

In this approach, the goal was to mimic the mechanical properties of tissue surrounding the native nerve while repeating observed electric fields near the wound site to promote neurite outgrowth. Present nerve guides comprised of poly (lactic-co-glycolic acid) (PLGA), collagen, or polyvinyl acetate (PVA) fall short of native nerve’s mechanical properties either due to insufficient elasticity or loss of mechanical properties due to early degradation [87–89]. Based on extended mechanical integrity matching native nerve tissue due to improved degradative properties, PGSA was selected (Table 1) [78, 79].

It has been shown that thermal, mechanical, and degradative properties of PGSA were influenced by condensation reaction time of PGS (for which increased time resulted in increased molecular weight) and acrylation percentage (determined by NMR) [78, 81]. Increased molecular weight decreased tensile strength, increased elongation to break, and increased the degradation rate [78, 81]. Increased acrylation resulted in decreased tensile strength, decreased elongation to break, increased young’s modulus, and decreased the degradation rate [78, 81]. Given the target application for nerve repair requires relatively slow degradation time to enable regeneration, shorter condensation time was chosen. PGS was acrylated to result in PGSA at 25, 30, 35, and 40% acrylation, which enabled evaluation of mechanical and degradative properties to fine tune polymer properties. Comparison of NMR and ATR-FTIR demonstrated the PGS and PGSA were as indicated in previous publications [78, 81] and NMR spectra were used to confirm the specified 0, 25, 30, 35, and 40% acrylations of PGSA. ATR-FTIR demonstrated no difference in the PGSA spectra with a smooth curve from 1590–1650 cm^−1^ in accordance with Ifkovits, et al. which demonstrated maximum reaction during photo polymerization [81].

The PGS x-ray diffractogram demonstrated an amorphous characteristic peak at ~20^o^, which is in accordance with the x-ray diffraction pattern published for PGS in Chen, et al. [90]. PGSA samples demonstrated the appearance of three peaks in the 2*θ* region occupied by the amorphous characteristic peak at ~20°. This development of peaks suggests a more ordered structure with acrylation and UV crosslinking. Comparison of x-ray diffraction patterns of DMPA [91] and UV-crosslinked polymers using DMPA [92–94] as a photo initiator to data in this work do not explain the emergence of the three peaks. Since NMR illustrated the acrylate peaks as key changes in PGSA vs PGS, these peaks may suggest acrylation resulted in a more ordered structure. The minor peak at 7.2° observed for PGS (heat cross-linked at 150 °C) and PGSA samples which were UV cross-linked was also observed by Chen, et al. for PGS heat cross-linked at 130 °C [90]. The recurrence of the minor peak at 7.2 °C may indicate increased order of the structure at higher crosslinking temperatures, as well as, for the UV cross-linked PGSA.

Glass transition temperatures decreased 1 to 2 °C per 5% acrylation but did not distinguish between higher acrylations (35 and 40%). These glass transition values are similar but slightly lower than those published with similar acrylations but with higher molecular weights. This may suggest that molecular weight also affects T_g_ [78]. In contrast, T_g_ for PGS-CinA (a variation of photo polymerizable PGS) decreased with increasing substitution of cinnamate [95]. All of the PGS, PGSA, and PGS-CinA polymers and copolymers were in a rubbery state at room temperature and at 37^o^C.

Moduli comparison demonstrated all of untreated and T2 treated PGS and PGSA samples were within range of native nerve tissue [Table 1]; [71, 72, 96] whereas for T1 only PGSA 40% met the range. In this work, the modulus of untreated PGSA 25% was ~60% of that of Ifkovits, et al., in which untreated PGSA at 22% acrylation was synthesized with additional time and an inhibitor step [97]. Comparison of T1 treated PGSA 30, 35, and 40% acrylation to Nijst, et al.’s PGSA at 30, 35 and 45% acrylations and treated similarly to T1 demonstrated our PGSA had average moduli from 70–95% that Nijst, suggesting sample similarity to prior work [78]. Moduli comparison to other forms of PGS acrylation, such as, PGS-cinnamate and PGS-methacrylate demonstrated our PGSA samples were 4–10x stiffer than PGS-cinnamate and 1.7x stiffer than PGS-methacrylate [95, 98]. PGSA modulus was 68–130x higher type I Collagen (composition for 50% of marketed nerve conduits) modulus, which indicated the PGSA materials were stiffer, which should aid in surgical handling.

Percent strain comparison showed all untreated and T2 treated PGS and PGSA samples, as well as, T1 PGSA 35% and 40% acrylations were within range of native nerve tissue. [Table 1] [71, 72, 96] Comparison of untreated PGSA 25% to Ifkovits’ PGSA 22% showed our materials withstood 2x the strain of Ifkovits’ PGSA [97]. The strain trend for Nijst’s ethanol treated PGSA increased up to 35% and then decreased at 45%, whereas our sample strains decreased with increasing acrylations independent of treatment type [78]. T2 PGSA samples were 30–50% of that of Nijst’s treated PGSA [78]. Strain comparison to PGS-cinnamate demonstrated PGSA samples were elongated 30% that of the PGSA-cinnamate. This may be partially explained by differences in mechanical testing parameters but does correlate to our stiffer material being less flexible [95]. PGSA strain compared to type 1 collagen was 25%, which also correlates to our stiffer material being less flexible.

Ultimate stress comparison of Nijst’s ethanol treated PGSA 25% was equivalent to T1 treated PGSA 25% and 25% that of T2 treated PGSA 25% [78]. This further reiterated structural damage from T1 vs T2. It is of note that untreated, T1, and Nijst samples demonstrated increasing stress with increased acrylation; however, for T2 samples the stress at break was not significantly different with increased acrylation. Comparison of ultimate stress showed that stresses for PGS and PGSA samples were at least 10x greater than collagen and 4x greater than PGS-cinnamate [95, 99].

Degradation properties demonstrated no significant difference in mass remaining across acrylations. Swelling diameter was significantly different for only for 25% vs. the other acrylations. Further, as the polymer lost mass, samples continued to swell. This indicated that degradation occurred due to a combined effect of surface erosion and swelling, which is in contrast to previous work, which suggested only surface erosion [78, 81]. The degradation properties met with expectations for our target conditions (Table 1) and the similarity in degradation for PGSA 35% and 40% further reinforced use of PGSA within the range 35–40%.

### Biological interactions with PGSA and PGSA/PPy composites

To promote neurite outgrowth, it was crucial to evaluate cellular growth on the developed materials in terms of attachment, alignment, and potential for molecular gradients. For some polymers, neuron growth/attachment has been limited or prevented due to the absence of added attachment proteins (e.g., fibronectin, fibrin, laminin, collagen, thrombin, heparin, poly-L lactide, and factor XIII) and/or peptides (e.g., IKVAV and RGD) [19, 32, 40, 84, 100–104]. The BCA assay results demonstrated the ability of both fibroblasts (HEPM) and neuronal (B35) cells to attach to PGSA with additional attachment proteins or peptides. Fibroblastic adhesion served as an indicator for general cellular adhesion, while neuronal cell adhesion suggested the material properties were suitable for specific application for peripheral nerve repair.

In addition to protein attachment, micro- and nano- textured topographies have been utilized by other researchers as an aid for cellular alignment [20, 84–86, 105]. In seeming contrast, the ridges in this study were perpendicular to the desired direction of nerve outgrowth to alternate polymer phases (Fig. 1). While alignment of cells has been facilitated using topography, linear cell growth has chiefly occurred due to synergistic effects of Schwann cells and/or attached surface proteins [85]. Further, neurites have demonstrated bridging across micropatterned grooves [84]. The neurites were observed extending to and connecting with other neurons through the low conductivity material with cellular alignment and processes progressing to cells in neighboring valleys, indicating dominance of the charged surfaces influencing directionality more than topography. This observation is in agreement with previous work which demonstrated that electric fields of physiological strength tend to dominate and override other directional cues [1, 53, 106]. Topography did not demonstrate a restriction to cell extension in this study and it appears that the conductivity differential increased proliferation with many cells extending axons.

Histological evaluation of the biphasic materials fashioned into tubes and implanted in a rta model of a sciatic nerve transection confirm that the biphasic materials can be utilized as suitable nerve conduits. There was a higher abundance of myelinated axons in the tubes made of biphasic material compared to the control tubes composed of silicone. Moreover, there was minimal fibrosis observed throughout the conduit, which is critical for allowing nerve regeneration throughout the tube. This acute study confirms that the biphasic materials are supportive of axon extension, even in the absence of electrical stimulation. No animals had complications from the implantation of the biphasic tubes, further supporting that additional experiments are justified to examine the application of electrical stimulation to the biphasic material in vivo.

### Electrical stimulation

The biphasic structure of the sheets in this study was designed to create repeated electric field gradients by alternating high and low electrically conductive materials connected to an external electrical stimulator. Previous studies have demonstrated that growth factor release coupled with low currents can create biologically significant linear cell migration responses in vivo [56, 59, 60, 70, 107]. Additionally, electrical stimulation from the proximal to distal ends of transected nerves has demonstrated promise [51, 61–65]. To separate influences, adhesive properties, substrate materials, and net surface charges enhanced cellular response to electric fields in vitro [54, 63, 64, 66–69]. Work combining conductive materials with stimulation and support cells (e.g., Schwann cells or olfactory ensheathing cells) has also shown great promise with components combined with electric stimulation producing additive benefit to neurite outgrowth and numbers, as well as, increased proliferation [60–62, 65]. Electrical stimulation studies consistently have shown positive cellular response utilizing a single electric field or with maintained stimulation along a continuously conductive substrate. We theorized that there is an optimal electric field for cell response and designed our biphasic sheets to mimic and repeat the electric field lengths which were shown to be optimal in vivo [56, 70].

To our knowledge, this is the first study to repeatedly alternate surface conductivity with and without external stimulation to assess in vitro cellular response. Since the goal of the electrical stimulation for an actual wound site is to stimulate growth across region, three days of stimulation evidenced the greatest migration and proliferation of the neuronal cell lines. Based on these data, 3 day stimulation would be the experimental group expected to perform best in vivo.

## Conclusions

In this study, we successfully synthesized PGSA with target mechanical and electrical properties needed for peripheral nerve repair. The prepared polymers were validated with chemical characterization from published work and demonstrated cell growth with no significant difference compared to control materials. Based on mechanical properties, comparison of treatment for cellular applications revealed gradated ethanol treatment was preferable to previously published concentrated ethanol treatment. Electrical stimulation results demonstrated that cellular migration was maximized with 3 days of stimulation. Based on these results, we prepared conduits composed of repeated high and low conductivity materials suitable for implantation in the rat sciatic nerve model for nerve repair, and demonstrated that they are superior to silicone conduits. These results suggest that biphasic conducting conduits may succeed in maintaining mechanical properties without inducing compression injuries while incorporating the added qualification of providing a guidance cue for directing nerve outgrowth. The results indicate that repeated electrical stimulation incorporated into a conduit device for nerve repair holds great promise as a new tool for accelerated peripheral nerve repair and extension of peripheral nerve repair to previously untreatable patients.
